# Test of the Deception Hypothesis in Atlantic Mollies *Poecilia mexicana*—Does the Audience Copy a Pretended Mate Choice of Others?

**DOI:** 10.3390/biology7030040

**Published:** 2018-07-13

**Authors:** Klaudia Witte, Katharina Baumgärtner, Corinna Röhrig, Sabine Nöbel

**Affiliations:** 1 Research Group of Ecology and Behavioral Biology, Department of Chemistry-Biology, Institute of Biology, University of Siegen, Adolf-Reichwein-Str. 2, 57076 Siegen, Germany; k.baumgaertner@gexaso.de (K.B.); C_roehrig-corinna@web.de (C.R.); 2 CNRS, Université Toulouse, IRD, UMR 5174, EDB (Évolution & Diversité Biologique), 118 Route de Narbonne, F-31062 Toulouse CEDEX 9, France; sabine.noebel@univ-tlse3.fr

**Keywords:** sexual selection, public information, male mate choice, female mate choice, audience effect, mate-choice copying, social learning, eavesdropping, Atlantic molly, *Poecilia mexicana*

## Abstract

Animals often use public information for mate-choice decisions by observing conspecifics as they choose their mates and then copying this witnessed decision. When the copier, however, is detected by the choosing individual, the latter often alters its behavior and spends more time with the previously non-preferred mate. This behavioral change is called the audience effect. The deception hypothesis states that the choosing individual changes its behavior to distract the audience from the preferred mate. The deception hypothesis, however, only applies if the audience indeed copies the pretended mate choice of the observed individual. So far, this necessary prerequisite has never been tested. We investigated in Atlantic molly males and females whether, first, focal fish show an audience effect, i.e., alter their mate choices in the presence of an audience fish, and second, whether audience fish copy the mate choice of the focal fish they had just witnessed. We found evidence that male and female Atlantic mollies copy the pretended mate choice of same-sex focal fish. Therefore, a necessary requirement of the deception hypothesis is fulfilled. Our results show that public information use in the context of mate choice can be costly.

## 1. Introduction

The evolution of mate choice is one of the most fascinating topics in biology. Since Darwin 1871 [1] proposed that females choose mates and are, therefore, the driving force of extravagant secondary sexual traits in males, the debate on how individuals choose mates and what kind of intrinsic and extrinsic factors shape these preferences is still relevant. Although most models in sexual selection assume that males and females exhibit genetically based mate preferences [2,3,4,5,6], it is widely accepted that the environment and, hence, non-genetic factors can shape mate choice as well. Mate-choice decisions can be altered by ecological factors [7,8,9], maternal effects [10] and by the social environment [11,12,13,14,15]. The social environment, especially in group living animals, provides opportunities to extract information by observing conspecifics and their interactions among each other and the environment [16,17,18]. In the context of mate choice, public information can be used to find and choose potential partners, to detect fertile females [19], to evaluate the quality of mating partners [11], or to assess conspecifics as potential rivals in mate choice [20,21]. 

One form of using public information in the context of mate choice is mate-choice copying (hereafter MCC) [22,23]. Males and females copy the mate choice of others by observing conspecifics during sexual interactions and then choosing or rejecting [24] the same individual or an individual of the same phenotype [25] as a mate observed earlier [23,26,27]. This is true, even when the conspecifics sexually interact at a distance [28]. When genetic information is in conflict with public information, it has been shown that the influence of socially acquired information on mate choice is stronger than genetically-based preferences for certain male phenotypes in females [29,30]. Females maintain a new mate preference learned via MCC and can serve as models for other females [31,32]. Several theoretical models of MCC emphasize the importance of this strategy in sexual selection and have investigated how copying could evolve and be maintained in a population [26,33,34,35,36,37,38,39,40]. MCC can even support the evolution of novel traits in a species [25,41,42]. Recently, a theoretical approach has indicated that MCC can play an important role in speciation and hybridization [43]. 

So far, MCC has been found in several taxa like mammals [44,45,46,47], birds [25,48,49,50,51,52,53,54,55], and in invertebrates [56,57,58]. However, most studies on MCC have been performed in fish, especially in Poeciliids [15]. Experimental studies on MCC were performed in guppies *Poecilia reticulata* [59,60,61,62,63,64], in the sailfin molly *Poecilia latipinna* [11,19,24,28,65,66,67,68,69], in the Atlantic molly *Poecilia mexicana* [69], in the humpback limia *Limia nigrofasciata* [70], in the Japanese medaka *Oryzias latipes* [71,72]; and in other fish like the three-spined sticklebacks *Gasterosteus aculeatus* [73], the white belly damselfish *Amblyglyphidodon leucogaster* [74], the ocellated wrasse *Symphodus ocellatus* [75], and the deep-snouted pipefish *Syngnathus typhle* [76]. Besides numerous lab studies on MCC, experimental field studies show that MCC is a biologically relevant mate-choice strategy [48,63,66,74,75]. Although most studies regarding MCC focus on females, there is good evidence that males copy the mate choice of others as well [19,21,64,66].

When animals observe other conspecifics during mate choice, it is possible that the observing individual (the audience) is detected by the interacting conspecifics. The presence of an audience can lead to a change in behavior of the observed individuals. This phenomenon is called audience effect. The audience effect is a wide-spread phenomenon and has been studied in various contexts (e.g., feeding [77], food caching [78], predator detection [79], and mate choice [80]). The audience effect has been studied in various taxa (insects [81,82], birds [83,84,85,86,87,88], mammals [78,79,89,90,91,92]), especially in fish: e.g., in the fighting fish *Betta splendens* [93,94,95], in the three-spine stickleback *G. aculeatus* [96], in the guppy *P. reticulata* [64,80], in the sailfin molly *P. latipinna* [97,98], and in the Atlantic molly *P. mexicana* [99,100,101].

In the Atlantic molly, Plath et al. [99,100] and Ziege et al. [101] found that males diminished or even reversed their initial preference for larger females and spent more time with smaller females in the presence of a conspecific audience male. The authors interpreted these results as meaning that males recognized the audience male as a competitor and tried to deceive the audience male about their real mating preference to avoid sperm competition, because surrounding audience males might copy the males’ mate choice. This deception hypothesis [100,102] is largely accepted. The deception hypothesis is based on the assumption that the audience will copy the deceptive mate choice of the choosing individual. This necessary prerequisite, however, has never been tested. 

Our aim is to test whether male and female Atlantic mollies (1) show an audience effect, by altering their initial mate-choice decision when a same sex audience is present, and (2) whether the audience fish that had just witnessed the mate choice of the focal fish copied that mate-choice decision. Thus, we combined the typical setup to test the audience effect with a MCC experiment. We used the Atlantic molly as a model species because males and females copy the mate choice of others [69,103], and both sexes show an audience effect although it is much weaker in females [99,100,101,104,105]. 

We predicted that focal fish would at least decrease their preference for their initially preferred mate in the presence of an audience fish and that the audience fish which had witnessed the altered mate choice would copy that mate choice. Our results provide support for the deception hypothesis in the context of the audience effect in mate choice. 

## 2. Materials and Methods

### 2.1. Study Species and Housing Conditions

Atlantic mollies are small neotropical fish inhabiting fresh and brackish water [106]. They are livebearers of the family Poeciliidae without parental care and live in mixed-sex shoals [69]. All fish used in our experiments were mature adults and descendants from a population from Tampico, Mexico, caught in 1995. Fish were approximately two years old. All fish were housed in mixed-sex groups in several large housing tanks (80 × 35 × 40 cm^3^), each with approximately 30 individuals, under a 14:10 h light-dark-cycle and a constant temperature of 26 ± 1 °C. For more detailed information, see Supplementary Material. Tanks were aerated, filtered and equipped with plants and gravel. Fish were fed daily with flake food (JBL GmbH & Co. KG, Neuhofen, Germany), frozen chironomid larvae, or frozen *Artemia* spec. One week before we started the experiments, we separated fish by sex and housed them under the same condition in three large tanks, two for the females and one for the males. During the testing period, fish used in experiments were separated in individual tanks (40 × 25 × 41 cm^3^) and kept under the same light regime and nutrition condition as in the home tanks. For the performed experiments, we used focal fish, audience fish, and stimulus fish. Focal fish and audience fish were of same sex within each experiment and could choose between two stimulus fish of the opposite sex successively. The audience fish was presented during the second mate-choice test of the focal fish. After finishing the experiments, we released all fish back into their home tanks. 

### 2.2. General Experimental Procedure 

**Figure 1:**
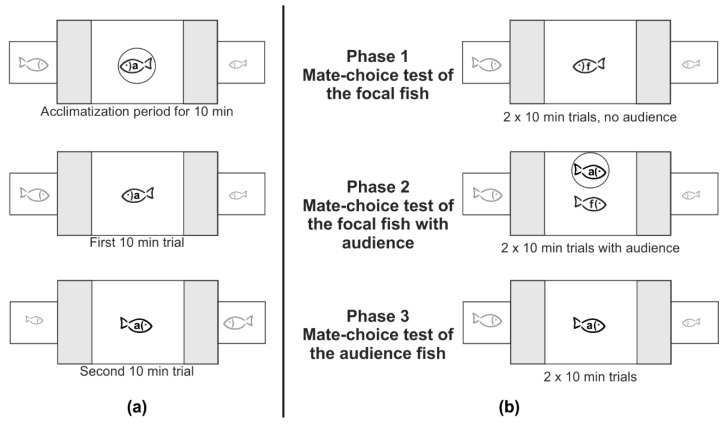
Setup of the (**a**) single mate-choice experiment of the audience fish on Day 1; and (**b**) setup and phases of the social mate-choice experiment of focal and audience fish on Day 2; audience fish: a, focal fish: f. Stimulus fish always differed in size by about 5 mm. Grey areas indicate mate-choice zones. Black vertical bars on the front glass of the test tank limited the grey areas and showed the position of glass sticks laying on the gravel. Circle indicates Plexiglas cylinder.

We performed male and female mate-choice experiments and controls in the same manner. We conducted single mate-choice experiments to determine the initial mate choice of audience fish, social mate-choice experiments to investigate the audience effect on focal fish and the MCC response of audience fish (see Figure 1), and controls to test for mate choice consistency. 

All experiments and controls were performed in a large test tank (100 cm × 50 cm × 40 cm) and two small stimulus tanks (20 cm × 25 cm × 40 cm) each adjacent to each end of the large tank (see Figure 1). All tanks had gravel on the bottom and were filled with water up to 35 cm. Water temperature was 24 ± 1 °C. The back and the sides of all tanks were covered with blue plastic foil leaving only a view from the front to allow observations and to avoid any disturbances from outside the tanks during experiments. Two fluorescent tubes (2 × 60 W) for illumination were placed centrally 100 cm above the setup. Before and after, but not during experiments, water was filtered and aerated by an air stone. We marked a mate-choice zone in the test tank 20 cm in front of each stimulus tank with black vertical bars on the front glass of the test tank and with two clear glass sticks laying in the test tank on the gravel 20 cm apart from both ends of the test tank. In the following, we describe in detail how we performed the experiments and the controls in general. Then we provide specific information for male and female mate-choice experiments. Because we needed for each test a focal fish and an audience fish with similar initial mate choice, we first determined the initial mate choice of the audience fish in a binary single mate-choice test. 

#### 2.2.1. Single Mate-Choice Experiment of the Audience Fish

The single mate-choice experiment was conducted one day before the social mate-choice experiment (see below). In the single mate-choice experiment, the fish later used as the audience fish in the social mate-choice experiment could choose between two stimulus fish of the opposite sex without any other conspecific present. First, we inserted opaque screens between the test tank and the two smaller tanks for stimulus fish. The audience fish was placed into the test tank and two stimulus fish (different in body length of at least 5 mm) were placed into the stimulus tanks one at each side of the test tank. The side on which the larger stimulus fish was presented was randomized. Each fish could explore and acclimatize to the new environment. After 20 min, the audience fish was gently placed into a clear Plexiglas cylinder (11 cm diameter) in the middle of the test tank, and the opaque screens were removed. During this period, the audience fish was able to observe both stimulus fish for 10 min (see Figure 1a). After this period, we released the audience fish out of the cylinder and a person (K.B. or C.R.), sitting 3 m away from the front of the test tank, recorded the time the audience fish spent within the 20 cm mate-choice zone in front of each stimulus tank with two stop watches, for 10 min. We then reinserted the opaque screens, placed the audience fish back into the Plexiglas cylinder in the middle of the test tank for 5 min, switched the stimulus fish between the two tanks and removed the opaque screens again. Then we released the audience fish again out of the cylinder and repeated the first mate-choice trial to test for side biases (see below). 

We measured the absolute association time (in seconds) an audience fish spent within each mate-choice zone by adding the time spent in the same mate-choice zone in both 10 min trials of the single mate-choice test. The audience fish was considered to prefer a particular stimulus fish if it spent more than 50% of the total association time within the mate-choice zone in front of that particular stimulus fish during the two 10 min mate-choice trials. This is a commonly used criterion to decide which of two stimuli was chosen [99]. Association time is a well-established measurement to determine mate choice in Poeciliids when no direct contact is provided [28,68,70,71,106,107]. Although association time is an indirect measurement of mate preference, several studies in different species of fish showed that the time females spent with a male was positively correlated with the probability of copulation with that same male [108,109,110,111,112]. 

An audience fish was considered to be side biased if it spent more than 90% of the total time in the same mate-choice zone in both 10 min trials although stimulus fish had been switched. Those audience fish were rejected from the analysis. This criterion has been used in several other studies with Poeciliids and other fish species [19,24,67,113,114]. 

#### 2.2.2. Social Mate-Choice Experiment

After finishing a single mate-choice experiment, we started with the social mate-choice experiment combining the audience effect and MCC. The social mate-choice experiment consisted of two phases; Phase 1 including the first and Phase 2 the second mate-choice test of the focal fish, and Phase 3 including a mate-choice test of the audience fish. The second mate choice test of the focal fish was performed in the presence of the audience fish (see Figure 1b). We would like to emphasize that we used different stimulus fish in the single mate-choice experiment and in the social mate-choice experiment. When comparing mate-choice decisions in audience fish, we compared a mate choice for the phenotype of the stimulus fish, not for a specific individual. 

Phase 1: 1. Mate-Choice Test of the Focal Fish

The first mate-choice test of the focal fish was performed without an audience fish and served to determine the initial mate choice of the focal fish. It was performed in the same manner as the single mate-choice test described in detail above (Figure 1). 

Phase 2: 2. Mate-Choice Test of the Focal Fish

After this first mate-choice test, we immediately started the second mate-choice test and tested whether the focal fish would change its mate-choice decision when the audience fish is present. Thus, we tested whether male and female Atlantic mollies showed an audience effect.

First, we inserted the opaque screens between the test tank and the stimulus tanks, placed the focal fish back into the Plexiglas cylinder for 5 min and placed an audience fish of the same sex and of similar body length as the focal fish into the large test tank so that the audience fish could explore the tank. After 5 min the audience fish was then gently placed into another clear Plexiglas cylinder (11 cm diameter) next to the focal fish. We removed the opaque screens and released the focal fish into the test tank and started the mate-choice test within Phase 2. We recorded the time the focal fish spent within the mate-choice zones in front of the same two stimulus fish as in the first mate-choice test, for two 10 min trials, including switching the stimulus fish after the first 10 min trial. We decided to present the audience fish always in the mate-choice test within Phase 2, because we needed to measure first the initial mate-choice decision of the focal fish when no conspecific was present to be able to compare its initial mate choice to the mate choice in the presence of an audience. We controlled for mate-choice consistency in focal fish (Control 1 and 2).

Phase 3: Mate-Choice Test of the Audience Fish

After finishing the mate-choice test in Phase 2 (Figure 1), we immediately started Phase 3 and tested whether the audience fish that had observed the mate choice of the focal fish from inside the Plexiglas cylinder, would copy the mate-choice decision of that focal fish. We first inserted the opaque screens between the test tank and the stimulus tanks again, removed the focal fish, and released the audience fish out of the Plexiglas cylinder into the test tank and removed the opaque screens. Now, the audience fish was allowed to choose between the same two stimulus fish as the focal fish before but without the presence of another same sex conspecific. To test whether the audience fish copied the mate choice of the focal fish we compared the mate choice of the audience fish in Phase 3 with its initial mate choice decision in the single mate-choice test and with the mate choice of the focal fish in Phase 2.

#### 2.2.3. Controls for the Social Mate-Choice Test

Control 1—Mate Choice Consistency in Focal Fish When no Audience was Present

Here, we investigated whether focal fish choose consistently in the mate-choice test in Phase 1 and 2 of the social mate-choice experiment when no audience was present in the Phase 2. We performed this control in the same setup under the same conditions and same procedures as the social mate choice experiment. The only difference was that the second clear Plexiglas cylinder in Phase 2 contained no audience fish. 

Control 2—Mate Choice Consistency in Focal Fish When the Audience Fish was Present but not Visible 

In this control we investigated whether focal fish chose consistently in the mate-choice test in Phase 1 and 2 when an audience fish was presented in Phase 2 but inside an opaque cylinder and thus not visible to the focal fish. We performed this control in the same setup under the same conditions and procedures as the social mate-choice experiment with one exception. We placed the audience fish directly into the opaque cylinder. It was not allowed to explore the tank. 

Control 3—Mate Choice Consistency in Audience Fish When no Public Information was Available

For this control we used data collected in Control 2 to test whether audience fish chose consistently and preferred the same mate-type (larger or smaller mate) when they had no opportunity to copy the mate choice of the focal fish while they were inside an opaque cylinder during the mate-choice test in Phase 2. For this we compared the mate-choice decision of audience fish in Control 2 with their initial mate-choice decision in the single mate-choice experiment.

#### 2.2.4. Measuring Fish Body Length

After each single mate-choice experiment, each social mate-choice experiment, and after each control we measured body lengths of all fish used in a test as the standard body length, from the tip of the snout to the end of the caudal peduncle in males and females. Stimulus fish used in a test always differed in body length by about 5 mm. Focal fish and audience fish used within a test were matched for body length. Standard body lengths of all used fish are given in Tables S1 and S2.

### 2.3. Male Mate-Choice Experiments

Each male was used only once as a focal male in the social mate-choice experiment. Each audience male was used once in the single mate-choice experiment and afterwards once in the social mate-choice experiment. Stimulus females were used twice, but with the second time always in combination with another female. In each control we used new focal males and audience males. In all experiments and controls, focal males and audience males used together in a test were matched for their initial mate choice, i.e., a mate choice for either larger or smaller females. Thus, we used an audience male with a preference for larger females in a social mate-choice test with a focal male expressing an initial preference for larger females as well. We tested 20 focal males and 29 audience males. We could use 20 focal males and had to exclude nine audience males because of side preferences or a mismatch in mate preferences. In Control 1, we tested 20 focal males. In control 2, we tested 21 focal males and used 20 audience males. One focal male had to be excluded because of side preferences. For Control 3, we used data from 20 audience males used in Control 2. 

### 2.4. Female Mate Choice Experiments

Each female was used only once as a focal female in the social mate-choice experiment. Each audience female was used once in the single mate-choice experiment and afterwards once in the social mate-choice experiment. Stimulus males were used twice but always the second time in combination with another male. In each control, we used new focal females and audience females, where required. In all experiments and controls, focal females and audience females used together in a test were matched for their initial mate choice, i.e., a mate choice for either a larger or smaller male. Thus, we used an audience female with a preference for larger males in a social mate-choice experiment with a focal female expressing an initial preference for larger males as well. We tested 26 audience females and 26 focal females. Twelve females had to be excluded because of side preferences or a mismatch in mate preferences. We could use 20 audience females and 20 focal females. In Control 1, we tested 23 focal females and excluded 3 because of side preferences, leaving 20 focal females. In Control 2, we tested 25 focal females and used 25 audience females. Ten females had to be excluded because of side preferences and mismatch in initial mate choice, leaving 20 focal females and 20 audience females. For Control 3, we used data of the 20 audience females tested in Control 2.

### 2.5. Ethical Statement

The performed experiments and handling of the fish were in line with the German Animal Welfare Act (Deutsches Tierschutzgesetz) and approved by the internal animal protection commissioner Dr. Urs Gießelmann, University of Siegen, and the national Veterinary Authority (Kreisveterinäramt Siegen-Wittgenstein, permit numbers: 53.6 55-05). 

### 2.6. Data Analysis

All analyses were conducted with the R software 3.4.0 (R Core Team 2017, Vienna, Austria). The mate-choice scores used in the linear model (LM) analysis were calculated as (time spent in front of the non-preferred stimulus fish in the second mate-choice test/total time spent in both mate-choice zones in the second test)−(time spent in front of the non-preferred stimulus fish in the first mate-choice test/total time spent in both mate-choice zones in the first mate-choice test). For focal fish, the first and second mate-choice test correspond to Phases 1 and 2 of the social mate-choice experiment, respectively (Figure 1b). For audience fish, they correspond to Day 1 of the single mate-choice experiment (Figure 1a) and to Phase 3 of the social mate-choice experiment (Figure 1b). This score was used to evaluate the change in mate choice due to public information in focal and audience fish between the mate-choice experiments and the controls. Mate-choice scores were tested for normality with a Shapiro-Wilk test and then analyzed in a linear model. We analyzed all successful replicates, i.e., replicates in which both audience and focal fish showed the same initial mate choice and had no side preference (200 tests with 120 focal fish and 80 audience fish out of 235 focal or audience fish used).

The starting model included treatment with two levels (informed/uninformed), the type of fish (audience/focal fish), and sex (male/female) as fixed effects. Informed in this context indicates that fish were able to gather public information (corresponding to the mate-choice experiments) while uninformed fish had no opportunity to observe conspecifics during mate choice (corresponding to the controls). All starting models included interactions between fixed effects. We applied a backward selection method using *p*-values, by dropping out non-significant effects, starting with the highest order interaction. We used Akaike Information Criteria [115] to determine the final model(s). The significance of fixed effects was tested using Wald Chi-square tests implemented in the analysis of variance (ANOVA) function of the car package [116]. With this mating score we tested the prediction that the change in preference for the non-preferred stimulus fish (i.e., the time spent with the non-preferred stimulus fish in the second mate-choice test) should be greater for informed than for uninformed (control) focal and audience fish. We tested the effect of potential confounding parameters on the change in mate choice in univariate tests and found no significant effect of body length of the audience or focal fish (audience fish LM: df = 1, F = 0.015, *p* = 0.902; focal fish LM: df = 1, F = 1.370, *p* = 0.244), size difference between audience and focal fish (LM: df = 1, F = 0.055, *p* = 0.815), size of stimulus fish (preferred: LM df = 1, F = 0.039, *p* = 0.845; non-preferred: LM df = 1, F = 0.759, *p* = 0.365) nor size difference between stimulus fish (LM: F = 0.127, *p* = 0.723).

To further evaluate the change in mate choice in focal and audience fish, a second mate-choice score was calculated to make comparisons within a test and between test phases. The second mate-choice score is based on the time (in seconds) focal and audience fish spent in front of one stimulus fish divided by the total time spent in both mate-choice zones. Thus, we obtained a score for the preferred and the non-preferred stimulus fish of each focal and audience fish tested in single mate-choice experiments, in social mate-choice experiments, and in all controls. Here, the predictions were that focal fish should decrease their preference for their initially preferred mate in the presence of an audience fish and that the audience fish should copy that mate choice. To test this, we compared the mate-choice scores for preferred and non-preferred stimuli used within and between tests using Wilcoxon matched-pairs tests, and we compared mate-choice scores for preferred stimulus fish between focal and audience fish using Mann-Whitney U tests. We used non-parametric tests because in this case the normality assumption was rejected for this mate-choice score. To control for body size differences, we tested whether fish used within a test differed in standard body length using a paired *t*-test (this analysis can be found in the supplements).

## 3. Results

### 3.1. General Analysis

The starting model of variables explaining the first mate-choice score included three main effects (i) treatment (informed/uninformed), (ii) type of fish (audience/focal fish), and (iii) sex (male/female), plus all possible interactions. We found a trend for treatment (LM: df = 1, F = 3.096, *p* = 0.08) and a significant interaction between type of fish (focal/audience fish) and sex (male/female) (LM: df = 1, F = 6.387, *p* = 0.012). For further analysis, we split the data set by the type of fish and analyzed focal and audience fish separately. For focal fish, we found a significant effect of the sex (LM: df = 1, F = 14.7, *p* < 0.001) and treatment (LM: df = 1, F = 0.397, *p* = 0.049) on the mate-choice score. If focal fish were informed, thus aware of the presence of an audience, this had no effect on males (LM: df = 1, F = 0.643, *p* = 0.426), but did have an effect on female focal fish (LM: df = 1, F = 4.182, *p* = 0.046). In audience fish, neither sex (LM: df = 1, F = 0.024, *p* = 0.878) nor treatment (LM: df = 1, F = 0.165, *p* = 0.686) had an effect on the mate-choice score. It is worth, however, to have a closer look at the mate-choice decision of focal and audience fish in the different situations. In the following, we present the analysis with the scores for preferred and non-preferred stimuli within the mate-choice tests of the social mate-choice experiments and controls. 

### 3.2. Male Mate-Choice Experiments

**Figure 2:**
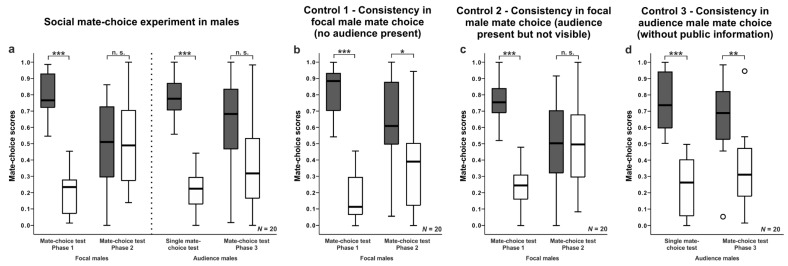
Male mate-choice experiment: Mate-choice scores of focal and audience males for preferred (grey boxplots) and non-preferred (white boxplots) stimulus females in single mate-choice experiments, in social mate-choice experiments, and controls. Boxplots show median, quartiles, and whiskers (1.5 x interquartile range). Circles indicate mild outliers. Significant *p*-values are from Wilcoxon matched-pairs tests (* *p* < 0.05; ** *p* < 0.01; *** *p* < 0.001; n.s. = non-significant).

Focal males showed a clear preference for one of the two females in Phase 1 (Wilcoxon matched-pairs test: N = 20, Z = −3.921, *p* < 0.001; see Figure 2a, Table S3). Seventeen of 20 audience males spent more time in front of larger females. In the mate-choice test of Phase 2 when the audience male was present, they showed no preference for one of the two females anymore (Wilcoxon matched-pairs test: N = 20, V = 115, *p* = 0.729; see Figure 2a and Table S3). They significantly decreased their time spent in front of the preferred female from the first to the second mate-choice test (Wilcoxon matched-pairs test: N = 20, V = 195, *p* < 0.001). Only 11 of 20 focal males spent more time in front of larger females. Thus, focal males altered their mate-choice decision in the presence of an audience male, and therefore, showed an audience effect.

Audience males showed a clear preference for one of the two females in the single mate-choice test (Wilcoxon matched-pairs test: N = 20, V = 210, *p* < 0.001; see Figure 2a and Table S3). Seventeen of 20 audience males spent more time in front of larger females. In Phase 3 after the observation of the altered mate-choice decision in focal males, they showed no preference for one of the two females anymore (Wilcoxon matched-pairs test: N = 20, Z = −1.469, *p* = 0.142; Figure 2a, Table S3). They significantly decreased the time spent in front of the preferred female phenotype from the first to the second mate-choice test (Wilcoxon matched-pairs test: N = 20, V = 158, *p* = 0.048). Only 13 of 20 audience males spent more time in front of larger females. Furthermore, they spent the same amount of time in front of the preferred female as the focal fish (Mann-Whitney U test: N = 40, W = 153, *p* = 0.208). Thus, audience males copied the altered mate choice of focal males.

Control 1—Mate Choice Consistency in Focal Males When no Audience was Present

Focal males showed a clear preference for one of the two females in the mate-choice test of Phase 1 (Wilcoxon matched-pairs test: N = 20, V = 210, *p* < 0.001; see Figure 2b and Table S3) and in the mate choice test in Phase 2 (Wilcoxon matched-pairs test: N = 20, V = 158, *p* = 0.048; see Figure 2b and Table S3). Thus, focal males chose consistently when no audience male was present and preferred the same female in both mate-choice tests, although they spent less time with the initially preferred stimulus female (Wilcoxon matched-pairs test: N = 20, V = 165, *p* = 0.005). In the mate-choice test of Phase 1, 16 of 20 focal males spent more time with larger females and in Phase 2, 13 of 20 focal males spent more time with larger females.

Control 2—Mate Choice Consistency in Focal Males when the Audience Male was Present but not Visible 

Focal males showed a clear preference for one of the two females in the mate-choice test of phase 1 (Wilcoxon matched-pairs test: N = 20, V = 210, *p* < 0.001; Figure 2c and Table S3). Sixteen of 20 model males preferred larger females. When audience males were present inside an opaque cylinder in Phase 2, however, focal males showed no preference for one of the two females anymore (Wilcoxon matched-pairs test: N = 20, V = 98, *p* = 0.92; Figure 2c and Table S3). In Phase 2, only 11 of 20 focal males preferred larger females. They significantly decreased the time spent in front of the preferred female phenotype from the first to the second mate-choice test (Wilcoxon matched-pairs test: N = 20, V = 183, *p* = 0.002). Thus, focal males did not choose consistently when an audience male was present but not visible to focal males.

Control 3—Mate Choice Consistency in Audience Males When no Public Information was Available

Audience males spent more time in front of a specific female phenotype in the single mate-choice experiment (Wilcoxon matched-pairs test: N = 20, V = 210, *p* < 0.001; see Figure 2d and Table S3) and in the mate-choice test of Phase 3 of the social mate-choice test when they had no opportunity to observe the mate choice of focal males (while inside of an opaque cylinder; Wilcoxon matched-pairs test: N = 20, V = 183, *p* = 0.002; Figure 2d and Table S3). In both mate-choice tests, 16 of 20 audience males preferred larger over smaller females. They preferred the same female phenotype in both mate-choice tests (Wilcoxon matched-pairs test: N = 20, V = 125, *p* = 0.475). Thus, audience males chose consistently between female phenotypes when they had no opportunity to copy. 

### 3.3. Female Mate-Choice Experiments

**Figure 3:**
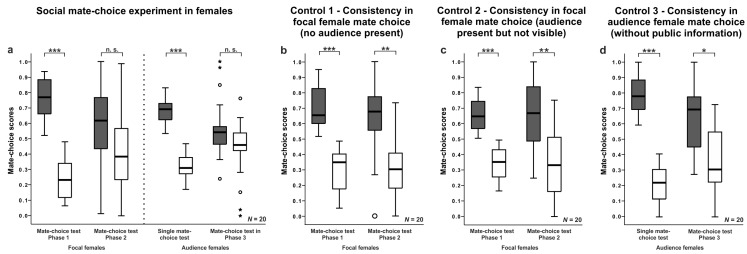
Female mate-choice experiment: Mate-choice scores of focal and audience females for preferred (grey boxplots) and non-preferred (white boxplots) stimulus males in single mate-choice experiments, social mate-choice experiments, and controls. Boxplots show median, quartiles and whiskers (1.5 x interquartile range). Circles indicate mild outliers, stars indicate extreme outliers. Significant *p*-values are from Wilcoxon matched-pairs tests (* *p* < 0.05; ** *p* < 0.01; *** *p* < 0.001; n.s. = non-significant).

Focal females showed a clear preference for one of the two males in the mate-choice test of Phase 1 (Wilcoxon matched-pairs test: N = 20, V = 210, *p* < 0.001; see Figure 3a, Table S4). Thirteen of 20 model females spent more time with larger males. In the mate-choice test of Phase 2 when the audience female was present, focal females showed no preference for one of the two stimulus males anymore (Wilcoxon matched-pairs test: N = 20, V = 152, *p* = 0.083; see Figure 3a, Table S4). Thus, focal females altered their mate-choice decision in the presence of an audience female and, therefore, showed an audience effect and tended to spend less time in front of the initially preferred male phenotype in the mate-choice test in Phase 2 (Wilcoxon matched-pairs test: N = 20, V = 154, *p* = 0.07 although 13 of 20 focal females still spent more time with larger males.

Audience females showed a clear preference for one of the two males in the single mate-choice test (Wilcoxon matched-pairs test: N = 20, V = 210, *p* < 0.001; Figure 3a, Table S4). Thirteen of 20 audience females spent more time with larger males. In the mate-choice test of Phase 3 after the observation of the focal female, they showed no preference for one of the two stimulus males anymore (Wilcoxon matched-pairs test: N = 20, V = 131, *p* = 0.153, Figure 3a, Table S4). They spent significantly less time in front of the initially preferred male phenotype from the first to the second mate-choice test (Wilcoxon matched-pairs test: N = 20, V = 166, *p* = 0.022). Now 16 of 20 audience females spent more time with larger males. Furthermore, they spent the same amount of time in front of the preferred male as the focal female in the mate-choice test before (Mann-Whitney U test: N = 40, W = 239.5, *p* = 0. 291). Thus, after observing the focal females’ mate choice, audience females changed their mate-choice decision and copied the altered mate choice of the focal female. 

Control 1—Mate Choice Consistency in Focal Females When no Audience was Present

Focal females showed a preference for one of the two male phenotypes in the mate-choice test of Phase 1 (Wilcoxon matched-pairs test: N = 19, V = 210, *p* < 0.001; see Figure 3b, Table S4) and in the mate-choice test of Phase 2 (Wilcoxon matched-pairs test: N = 19, V = 173, *p* = 0.002; see Figure 3b, Table S4). They spent the same amount of time in front of the initially preferred male from the first to the second mate-choice test (Wilcoxon matched-pairs test: N = 19, V = 121, *p* = 0.571). Twelve of 19 focal females spent more time with larger males in Phase 1 and in Phase 2. Thus, focal females chose consistently when no audience female was present. 

Control 2—Mate Choice Consistency in Focal Females When the Audience Female was Present but not Visible 

When audience females were present inside an opaque cylinder and thus not visible to the focal female in the mate-choice test of Phase 2, focal females spent significantly more time with a specific male phenotype in the mate-choice test in Phase 1 (Wilcoxon matched-pairs test: N = 20, V = 210, *p* < 0.001; Figure 3c, Table S4) and in the mate-choice test in Phase 2 (Wilcoxon matched-pairs test: N = 20, V = 174, *p* = 0.011; Figure 3c, Table S4). They showed a similar preference from the first to the second mate-choice test (Wilcoxon matched-pairs test: N = 20, V = 105, *p* = 1). In phase 1, 15 of 20 focal females spent more time with larger males, and in Phase 2, 16 of 20 focal females preferred larger males.

Thus, focal females chose consistently when an audience female was present but not visible to focal females.

Control 3—Mate Choice Consistency in Audience Females when no Public Information was Available

Audience females preferred a specific male phenotype in the single mate-choice test (Wilcoxon matched-pairs test: N = 20, V = 210, *p* < 0.001; Figure 3d, Table S4) and in Phase 3 of the social mate-choice experiment when they had no opportunity to observe the mate choice of focal females (while inside of an opaque cylinder; Wilcoxon matched-pairs test: N = 20, V = 162, *p* = 0.033; Figure 3d, Table S4), although they spent less time in front of the initially preferred male phenotype in the mate-choice test in Phase 3 (Wilcoxon matched-pairs test: N = 20, V = 169, *p* = 0.015). Fifteen of 20 audience females spent more time with larger males in Phase 1 and 13 females in Phase 2. Thus, audience females chose consistently between male phenotypes when they had no opportunity to copy.

## 4. Discussion

Our results showed that male and female Atlantic mollies altered their mate-choice decision when a same sex audience fish was present. Thus, male and female Atlantic mollies showed an audience effect. Subsequently, when male and female audience fish observed this altered mate-choice decision, male and female audience fish changed their initial mate-choice decision by copying the altered mate choice of the focal fish. Thus, misleading/deceiving others is an adaptive strategy in manipulating the mate-choice decisions of potential competitors and might increase own reproductive success. Based on our analyses, we found a trend for the type of treatment and a significant interaction between type of fish (focal/audience fish) and sex. For focal fish, we found a significant effect of the sex and treatment on the mate-choice decision. If focal fish were aware of the presence of an audience fish, this had no effect on males, probably due to the inconsistency in control 2, but we found a clear effect in female focal fish. In audience fish, neither sex nor treatment had an effect on the mate-choice decision. Thus, our results were not always clear-cut, but this might be due to the complex test situation and due to the fact that mate-choice behavior including public information is very sophisticated and many factors affected the behavior of the fish during the experiments, like familiarity with stimulus fish between tests and decrease of interest to stimulus fish. Nevertheless, focal fish clearly altered their mate-choice decision when an audience was present, and audience fish copied the altered mate choice of the focal fish, and thus exhibited a different mate choice than without observing the mate choice of the focal fish. 

To our knowledge, this is the first experimental evidence supporting the deception hypothesis in the context of the audience effect in mate choice. Makowicz et al. [80] showed in guppy males that focal males exhibited an audience effect and audience males displayed the same preferences as model males directly after the observation, but not after 24 h. Makowicz et al. [80], however, did not test the initial mate choice of audience males before the observation. Thus, it is impossible to decide whether audience males had copied the mate choice of focal males and whether the change in mate choice was due to the misleading information or due to inconsistency in mate choice. Auld and Godin [64] tested the audience effect and MCC in guppy males in two separate experiments. Herewith, they could only conclude that guppy males exhibit an audience effect and guppy males copy mate choices of other males in general. They could not conclude that audience males copied the deceptive mate-choice of focal males. 

It is known that *P. mexicana* females have genetically based mate preference for larger males [117], and similarly males always prefer larger females [118]. However, in the presence of a same sex audience, males and females seem to hide this genetically based preference and alter their mate-choice decision. The manner of performing the audience effect, however, might differ. In our study, focal females diminished their initial mate choice and showed no preference for one of the stimulus males in the presence of an audience female. Whereas in the study by Plath et al. [105], Atlantic molly females spent more time in the neutral zone when an audience female was present but did not change their mate-choice decision.

Deception is quite common in the animal kingdom and is described in various contexts such as in the case of stealing food [119,120,121] and false alarm calls to scare other individuals and mating. Male barn swallows *Hirundo rustica* used deceptive alarm calls when their mates left the nest vicinity during their fertile period, to cheat surrounding males and to lower the risk of extra-pair copulations [122]. The case of the pied flycatcher is a prominent example of deception. Males which have already established a territory and are paired, set up a second nesting territory several hundred meters apart from the first territory. They sing at the new nesting site and try to attract another female. As soon as the second female has laid eggs, the male leaves the second female alone and returns to his first mate to help feed the young [123]. In an experimental setup, Alatalo et al. [124] could further exclude alternative hypotheses and confirm the deception hypothesis that males deceive females about their actual mating status and gain extra offspring. 

Bierbach et al. [21] showed in the Atlantic molly that focal males react specifically to audience males that are either familiar or unfamiliar to focal males. Focal males exhibited a stronger audience effect to unfamiliar males than to familiar males. When focal males had witnessed familiar audience males as sexually active, they altered their mate-choice decision in the presence of the audience. This was not the case when focal males had witnessed the familiar male as sexually inactive beforehand. Thus, Atlantic molly males can remember other males and use relevant information they received about these potential competitors. In another study, Bierbach et al. [104] found that the personality of focal males affects the audience effect. Bolder males showed a stronger audience effect under high competition than shy males. In the sailfin molly, Nöbel and Witte [98] showed that males respond specifically to potential competitors as well. On the one hand, males reduced courtship displays towards the female partner when an audience male was present, but on the other hand, males transferred more sperm to the female partner in the presence of an audience male. This is the first study showing that public information use can be transferred into fitness investment measured as the amount of sperm.

The deception hypothesis in the mate-choice context suggests that males deceive other males to reduce risk of sperm competition for the deceiver [99,100,101]. Sperm competition in male Poeciliids is high (reviewed in [125]). Thus, deception is an adaptive strategy for males because the audience copies the pretended mate choice. Females may also benefit by misleading other females about their mate choice, because in Poeciliids, males often copulate with several females. Misled females will, therefore, reduce the risk of sperm depletion in the preferred male for the focal female. Competition in Poeciliid females can also affect female mate choice when females compete over males because number of (high quality) males is limited [126] or aggression between females is high [127].

Castellano et al. [102] explored the deception hypothesis in a game-theoretic model with two players, a focal male and an audience male, exploring under what circumstances males should exhibit an audience effect and mislead other males. The model showed that when costs are very high for focal males being copied, i.e., when potential females differ in quality, focal males should provide misleading information to the audience male. Thus, the model states that males are expected to cheat other males only in restricted conditions. 

An alternative to the deception hypothesis explaining the audience effect in the context of mate choice is the ‘split-attention hypothesis’ [99]. The ‘split-attention hypothesis’ suggests that the presence of an audience generally renders accurate mate choice a difficult task. Thus, animals cannot choose accurately between potential mating partners. This hypothesis however, seems to be unlikely in Atlantic mollies because in the natural habitat there are a lot of distractions from other individuals and there are always shoal mates around. If the ‘split-attention hypothesis’ is true, mate choice in mollies would be simply by chance. 

Another alternative to the deception hypothesis explaining the audience effect in the context of male mate choice might be male competition for mates. Here, focal males alter their mate choice to adjust their reproductive success to the currently perceived social competitive context [128,129]. In experiments testing the effect of competition, the focal male can first choose between two females. Afterwards, another male, the ‘competitor’, is presented directly next to the female the focal male has previously preferred. Thus, the focal male witnesses the ‘competitor’ associating with his initially preferred female. In this situation it might seem to the focal male that the other male, the ‘competitor’, has already chosen that female. Thus, it might be the best strategy for the focal male to alter his mate choice and spend more time with the previously non-preferred female to avoid sperm competition [128]. In our social mate-choice experiment, however, the audience male is not in direct vicinity to one of the two females. Instead the audience male is about 60 cm from each female and it might seem to the focal male that the ‘competitor’ has not made a mate-choice decision yet. Thus, this is a different situation for the focal male and he is able to mislead the audience male by altering his own mate choice as shown in our experiments. Thus, adjustment of the reproductive success is not an alternative to the deception hypothesis. 

For testing our prediction, it was absolutely necessary that we always tested first a focal fish without an audience and secondly with an audience. This, however, is in conflict with an order effect. It could be that in general same fish behave differently in the second mate-choice test than in the first one independently of the choice situation. The Controls 1 and 3 in male experiments and the Controls 1–3 in female experiments provide evidence that in general males and females chose similarly in two subsequent tests without any change in test situation.

Results from our controls showed that focal males and focal females chose consistently when no same sex audience fish was present during their mate choice (Control 1), and that audience fish chose consistently when they had not witnessed the mate choice of focal fish (Control 3). Interestingly focal males altered their mate-choice decision in Control 2, when an audience male was inside an opaque cylinder, and thus present but not visible to the focal male. We assumed that the focal male was probably able to perceive olfactory cues of the audience male in the opaque cylinder. In general, males use chemical cues to test whether females are receptive by nipping at the females’ genital opening [130]. Furthermore, chemical cues are important for species recognition in Poeciliids [131,132,133]. Because the cylinder stood on a layer of gravel in our test tank, water and chemical cues inside the cylinder could diffuse into the test tank through little holes between the edge of the cylinder and the gravel. To test this idea, we injected red food coloring (WUSITTA, Erich Wutzig GmbH, Sitzendorf, Germany) into the water inside the opaque cylinder in the large test tank standing on gravel like in the control. After a few seconds, red food coloring diffused at the bottom out of the cylinder and spread through the water of the large tank (see Figure S1). With this additional test, we could show that not only visual cues, but also olfactory cues might provide important information to the focal male about the presence of another male, a potential ‘competitor’, which results in an altered mate choice in focal males even when the presented audience male was not visible to the focal male. 

## 5. Conclusions

We tested the deception hypothesis in the context of the audience effect in mate choice, and found that both male and female Atlantic mollies alter their mate choice in the presence of a same sex audience fish and mislead the audience fish who copied the altered mate choice. Our study shows how complex the social information network in the Atlantic molly can be and that both sexes use public information for their mate-choice decisions. To our knowledge, this is the first experimental evidence supporting the deception hypothesis in the context of the audience effect in mate choice. Thus, the audience effect can be an adaptive strategy to lead a competitor away from the preferred mate in both sexes. Further experiments are necessary to explore how, when, and why fish use public information in mate choice, and how the use of public information affects sexual selection. 

## Supplementary Materials

The following are available online at http://www.mdpi.com/2079-7737/7/3/40/s1, Table S1: Standard body length (mm) of individuals used in male mate-choice tests given as median (1./3. quartile), Table S2: Standard body length (mm) of individuals used in female mate-choice tests given as median (1./3. quartile), Table S3: Absolute Time (s) spent of males in mate-choice zones in front of stimulus females, Table S4: Absolute Time (s) spent of females in mate-choice zones in front of stimulus males, Figure S1: Red food coloring diffused at the bottom out of the cylinder and spread through the water of the large tank.
