# Supplementary material for: Test of the Deception Hypothesis in Atlantic Mollies Poecilia mexicana—Does the Audience Copy a Pretended Mate Choice of Others?
Source: Biology (Basel). 2018 Jul 13;7(3):40. doi: 10.3390/biology7030040 (PMC6164261; doi:10.3390/biology7030040)
Supplement: Supplementary file 1 [file biology-07-00040-s001.pdf]

Additional information about stock tanks

All fish were housed in a large (approx. 46.8 m^2) room without windows. The room temperature was centrally controlled at 24–27 °C (humidity 50–60%), so that the water temperature in tanks was 22–25 °C. To provide best water quality, water was first filtered by a reverse-osmosis system and then minerals (Preis Diskus Mineralien, Bayerfeld, Germany) were added to achieve a conductivity of 200 µS/cm. The tanks were illuminated by two rows of twelve 125 cm long fluorescent lightings including UV-range (60 Watt, LZ 58), 2–12 cm above the tanks, which provided a light-dark cycle of 14:10 h.

All fish were separated by species and population and housed in mixed-sex shoals in large stock tanks (80 cm × 35 cm × 40 cm, water level 35 cm). All tanks contained a 3 cm gravel layer on the ground and several plants like *Aponogeton* spec, *Vallisneria* spec and *Lemna minor*. Water was cleaned by a filter (Hydor, Prime 20, Bassano del Grappa, Italia) and an UV-lamp (Tetra tec UV 400, Melle, Germany) and was additionally aerated by an air stone. The pH of the water was 7–8 and the conductivity was 400–500 µS/cm. Twenty litres of each tank were changed weekly. Every second week a teaspoon of salt (Ursalz, Erntesegen, Radolfzell, Germany) and every six weeks a handful of mussel lime were added to each tank. All fish were fed twice a day with flake food (Novo Bel, JBL, Neuhofen, Germany), frozen chironomid larvae or frozen *Artemia* spec.

Table S1. Standard body length (mm) of individuals used in male mate-choice tests given as median (1./3. quartile).

				**Single Mate Choice Experiment**			**Social Mate Choice Experiment**		
	**Focal Males**	**Audience Males**	**Paired t-test**	**Large Stimulus Females**	**Small Stimulus Females**	**Paired t-test**	**Large Stimulus Females**	**Small Stimulus Females**	**Paired t-test**
**Male mate choice experiments**	33.0 mm (30.0 mm / 37.75 mm) (N = 20)	31.5 mm (30.25 mm / 35.75 mm) (N = 20)	t = 0.96864; P = 0.345	54.0 mm (51.0 mm / 57.0 mm) (N = 19, one data-point missing)	30.0 mm (27.0 mm/41.5 mm) (N = 20)	t = 10.704; P < 0.001	52.0 mm (46.0 mm / 55.0 mm) (N = 20)	30.0 mm (27.0 mm / 41.0 mm) (N = 20)	t = 10.045; P < 0.001
**Control 1 for mate-choice consistency in focal males**	35.0 mm (33.0 mm / 39.0 mm) (N = 20)	-	-	-	-	-	52.0 mm (49.25 mm / 54.75 mm) (N = 20)	33.0 mm (32.0 mm / 38.0 mm) (N = 19)	t = 13.387; P < 0.001
**Control 2 for mate-choice consistency in focal males when audience males were inside an opaque cylinder**	34.0 mm (33.0 mm / 39.5 mm) (N = 20)	34.0 mm (33.0 mm / 37.75 mm) (N = 20)	t = 1.5771; P = 0.131	50.5 mm (42.0 mm / 56.25 mm) (N = 20)	33.5 mm (30.0 mm / 37.5 mm) (N = 20)	t = 10.455; P < 0.001	53.0 mm (52.0 mm / 59.0 mm) (N = 20)	33.0 mm (32.0 mm / 41.0 mm) (N = 20)	t = 7.5862; P < 0.001
**Control 3 (see Control 2)**									

Table S2. Standard body length (mm) of individuals used in female mate-choice tests given as median (1. /3. quartile).

				Single Mate-Choice Experiment			Social Mate Choice Experiment		
	**Focal Females**	**Audience Females**	**Paired t-test**	**Large Stimulus Males**	**Small Stimulus Males**	**Paired t-test**	**Large Stimulus Males**	**Small Stimulus Males**	**Paired t-test**
**Female mate choice Experiments**	38.5 mm (33.5 mm / 45.25 mm) (N = 20)	40.0 mm (34.0 mm / 48.0 mm) (N = 20)	t = -1.9494; P = 0.067	38.0 mm (35.0 mm / 40.0 mm) (N = 20)	30.0 mm (30.0 mm / 32.0 mm) (N = 20)	t = 13.608; P < 0.001	37.5 mm (35.0 mm / 39.0 mm) (N = 20)	30.0 mm (29.0 mm / 32.75 mm) (N = 20)	t = 14.416; P < 0.001
**Control 1 for mate choice consistency in focal females**	36.0 mm (38.0 mm / 46.0 mm) (N = 19)	-	-	-	-	-	38.0 mm (36.0 mm / 40.0 mm) (N = 19)	31.0 mm (31.0 mm / 32.0 mm) (N = 19)	t = 15.534; P < 0.001
**Control 2 for mate-choice consistency in focal females when audience females were inside an opaque cylinder**	36.0 mm (33.0 mm / 42.75 mm) (N = 20)	37.5 mm (34.25 mm / 39.75 mm) (N = 20)	t = 0.075434, P = 0.941	37.5 mm (35.0 mm / 39.0 mm) (N = 20)	30.0 mm (30.0 mm / 31.75 mm) (N = 20)	t = 15.874; P < 0.001	39.0 mm (38.0 mm / 40.0 mm) (N = 20)	31.0 mm (30.0 mm / 33.0 mm) (N = 20)	t = 15.947; P < 0.001
**Control 3 see Control 2**									

Table S3. Absolute Time (s) spent of males in mate-choice zones in front of stimulus females.

	**1. Mate-Choice Test**			**2. Mate-Choice Test**		
	**Preferred Females**	**Non-Preferred Females**	**Wilcoxon Test**	**Preferred Females**	**Non-Preferred Females**	**Wilcoxon Test**
Male mate choice experiments						
**Focal males (N = 20)**	760. 5 s (651 s / 930.8 s)	199.5 s (64.5 s / 288.5 s)	Z = −3.921, P < 0.001	155.5 s (76.3 s / 311.8 s)	146 s (119 s / 391.5 s)	Z = −0.355, P = 0.723
**Audience males (N = 20)**	743 s (614.5 s / 888.3 s)	212 s (127.5 s / 297 s)	Z = −3.922, P < 0.001	545.5 s (398 s / 764.5 s)	275 s (151.5 s / 399.3 s)	Z = −1.469, P = 0.142
Control 1 for mate-choice consistency in focal males						
**Focal males (N = 20)**	7771.5 s (451.3 s / 922.3 s)	109 s (54.8 s / 245.8 s)	Z = −3.923, P < 0.001	506.5 s (229 s / 705.5 s)	338 s (78.8 s / 473.5 s)	Z = −1.980, P = 0.048
Control 2 for mate-choice consistency in focal males when audience male inside an opaque cylinder						
**Focal males (N = 20)**	680 s (606 s/849.5 s)	229 s (166.8 s/273 s)	Z = −3.923, P < 0.001	556 s (256.5 s/612 s)	463 s (220.5 s/599.8 s)	Z = −0.109, P = 0.913
**Audience males (N = 20)**	779.5 (558.8 s/963.8 s)	291.5 s (58.5 s/397.3 s)	Z = −3.825, P < 0.001	622 s (550.5 s/858.8 s)	285 s (163.5 s/524 s)	Z = −2.913, P = 0.004

Table S4. Absolute Time (s) spent of females in mate-choice zones in front of stimulus males.

	1. Mate-Choice test			2. Mate-Choice test		
	**Preferred Males**	**Non-Preferred Males**	**Wilcoxon Test**	**Preferred Males**	**Non-Preferred Males**	**Wilcoxon Test**
			Female mate choice experiments			
**Focal females (N = 20)**	732.5 s (572.3 s / 872.3 s)	239.5 s (105.8 s / 327.5 s)	Z = −3.921, P < 0.001	206.5 s (99.3 s / 504.8 s)	154 s (53.8 s / 259.3 s)	Z = −1.680, P = 0.093
**Audience females (N = 20)**	617.5 s (545.5 s / 714.8 s)	282 s (235 s / 339.8 s)	Z = −3.924, P < 0.001	520.5 s (306.8 s / 616.3 s)	421 s (215.5 s / 522.5 s)	Z = −1.450, P = 0.147
			Control 1 for mate-choice consistency in focal females			
**Focal females (N = 19)**	620 s (436 s / 788 s)	277 s (155 s / 347 s)	Z = −3.824, P < 0.001	706 s (487 s / 703 s)	232 s (109 s / 399 s)	Z = −3.293, P < 0.001
			Control 2 for mate-choice consistency in focal females when audience inside an opaque cylinder			
**Focal females (N = 20)**	595 s (480.5 s / 773.5 s)	323.5 s (239.5 s / 407.3 s)	Z = −3.923, P < 0.001	575 s (392.3 s / 870.5 s)	293 s (150.5 s / 558 s)	Z = −2.570, P = 0.010
**Audience females (N = 20)**	769.5 s (596.3 s / 874 s)	185.5 s (114.3 s / 295.8 s)	Z = −3.921, P < 0.001	580 s (473.8 s / 666.8 s)	311.5 s (200.3 s / 608 s)	Z = −2.256, P = 0.024

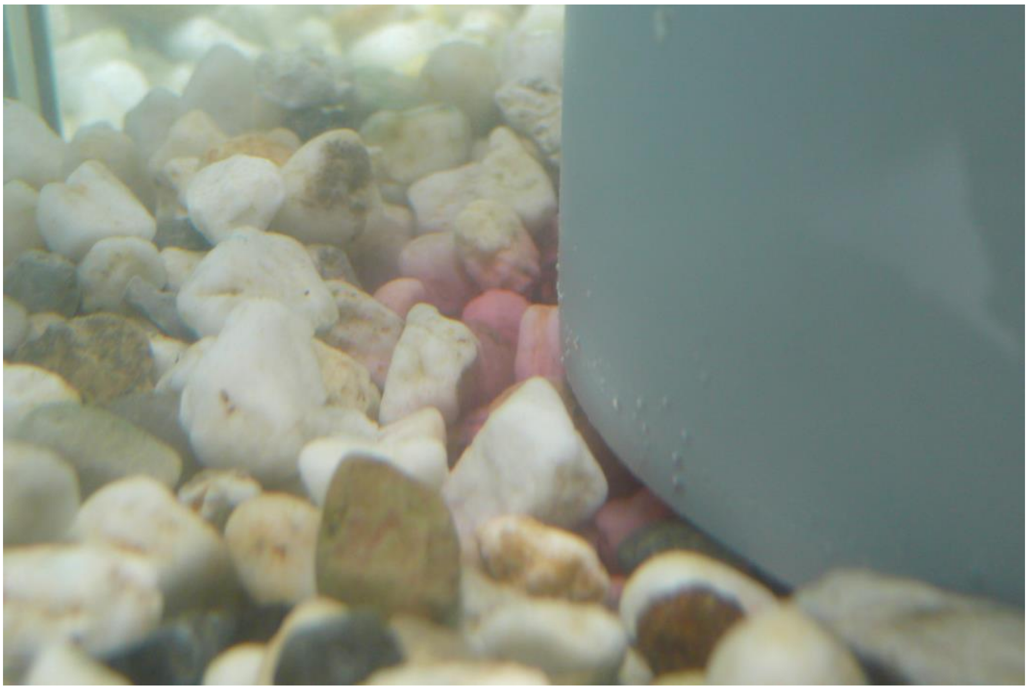

Figure S1. Red food coloring diffused at the bottom out of the cylinder and spread through the water of the large tank.
